# Higher risk of postpartum phase transition to immune-active among HBeAg-positive pregnant women with indeterminate phase

**DOI:** 10.3389/fcimb.2025.1652690

**Published:** 2026-01-07

**Authors:** Qiao Tang, Chun-Rui Wang, Hu Li, Zhi-Wei Chen, Xiao-Qing Liu, Yun-Ling Xue, Yue Qiu, Nan Cai, Yi Zeng, Peng Hu

**Affiliations:** 1 Department of Infectious Diseases, The Second Affiliated Hospital of Chongqing Medical University, Chongqing, China; 2 Institute for Viral Hepatitis, The Key Laboratory of Molecular Biology for Infectious Diseases, Chinese Ministry of Education, The Second Affiliated Hospital of Chongqing Medical University, Chongqing, China

**Keywords:** gray zone, immune-tolerance, inactive carrier, liver disease progression, natural history, chronic hepatitis B

## Abstract

**Background:**

Understanding the natural history of hepatitis B virus (HBV) infection helps determine the optimal timing of antiviral therapy. However, a gap exists in the literature regarding the dynamics of the natural history of HBV infection in pregnant women with chronic hepatitis B (CHB). This study aimed to explore the natural history of HBV infection during pregnancy and the postpartum period.

**Methods:**

We conducted a retrospective–prospective real-world study involving 276 pregnant women with CHB. Infection dynamics during pregnancy were characterized in 228 hepatitis B e-antigen (HBeAg)-positive and 48 HBeAg-negative participants, respectively, and during postpartum follow-up in 108 HBeAg-positive and 21 HBeAg-negative participants. HBeAg-positive participants received short-term antiviral intervention according to current guidelines. Liver disease progression was also evaluated.

**Results:**

Throughout pregnancy, the proportion of patients in the immune tolerance (IT) phase increased progressively, whereas the proportions of patients in the HBeAg-positive indeterminate phase (IP) and HBeAg-positive immune-active (IA) phase decreased. In the third trimester, the IT phase was dominant (48.7%), followed by the HBeAg-positive IP phase (24.6%), the HBeAg-positive IA phase (9.4%), the inactive carrier (IC) phase (9.4%), the HBeAg-negative IP phase (7.1%), and the HBeAg-negative IA phase (0.9%). During the postpartum period, a numerically higher cumulative incidence of phase maintenance (p = 0.150) and a significantly lower cumulative incidence of transition to the HBeAg-positive IA phase (p < 0.001) were observed in pregnant women in the IT phase compared with those in the HBeAg-negative IP phase. The cumulative incidence of phase maintenance (p = 0.900) and transition to the HBeAg-negative IA phase (p = 0.560) were comparable between pregnant women in the IC phase and those in the HBeAg-negative IP phase. The risk of postpartum liver disease progression was low among pregnant women across all disease phases.

**Conclusion:**

Pregnancy may have a pronounced impact on the dynamics of HBV infection in HBeAg-positive patients, particularly those in the IP phase at 24–28 weeks of gestation. Close postpartum monitoring is therefore warranted for this specific population.

**Clinical trial registration:**

## Introduction

Chronic hepatitis B (CHB), one of the leading causes of cirrhosis and hepatocellular carcinoma, remains a major public health problem worldwide (Terrault et al., 2018). The natural history of hepatitis B virus (HBV) infection is a major focus in CHB research, as it helps determine the stage of liver disease progression and guides the initiation of antiviral therapy (Lampertico et al., 2017; Terrault et al., 2018; You et al., 2023). According to the 2018 American Association for the Study of Liver Diseases (AASLD) guidelines, chronic HBV infection can be classified into four phases: the immune-tolerant (IT) phase (also known as HBeAg-positive chronic infection), the hepatitis B e-antigen (HBeAg)-positive immune-active (IA) phase (also known as HBeAg-positive chronic hepatitis), the inactive carrier (IC) phase (also known as HBeAg-negative chronic infection), and the HBeAg-negative IA phase (also known as HBeAg-negative chronic hepatitis) (Terrault et al., 2018). Numerous previous studies have investigated the dynamics of HBV infection and liver disease progression, including liver cirrhosis and hepatocellular carcinoma, in the general population across these phases (Andreani et al., 2007; Huang et al., 2022; Xu et al., 2023).

Pregnant women with CHB represent a subpopulation of particular clinical interest. Current guidelines recommend that prophylactic antiviral therapy be administered to pregnant women who are HBeAg-positive or have a high viral load to prevent mother-to-child transmission, but that treatment be discontinued at delivery or within 12 weeks postpartum (Lampertico et al., 2017; Terrault et al., 2018; You et al., 2023). Hormonal changes and immune alterations occurring during pregnancy and the postpartum period may lead to alanine aminotransferase (ALT) flares after delivery, and viral rebound may occur following treatment discontinuation (Wang et al., 2021; Song et al., 2022; Zhang et al., 2023). Pregnancy constitutes a unique physiological state characterized by profound immunological modulation and marked hormonal fluctuations, primarily aimed at maintaining fetal tolerance (Song et al., 2022; Zhang et al., 2023). These adaptations may distinctly influence host–virus interactions, potentially altering the natural history of HBV in ways not observed in non-pregnant individuals. In addition, several studies have reported relatively high rates of postpartum HBeAg clearance. Taken together, the natural history of HBV infection may be influenced by pregnancy, delivery, antiviral therapy, and treatment discontinuation. Despite these interactions, longitudinal data specifically describing dynamic changes across different phases of HBV infection during pregnancy and the postpartum period remain limited, representing a critical knowledge gap in the management of CHB during pregnancy and the postpartum period.

To address this gap, we conducted a retrospective–prospective real-world study to investigate the dynamics of HBV infection in women with CHB during pregnancy and after delivery, as well as postpartum liver disease progression among patients in different disease phases. We hypothesized that divergent postpartum outcomes in the natural history of HBV infection arise from underlying differences in infection characteristics and immune environments between HBeAg-positive and HBeAg-negative pregnant women.

## Patients and methods

### Ethical statement

This study was approved by the Ethical Committee of the Second Affiliated Hospital of Chongqing Medical University and registered in the Chinese Clinical Trial Registry (ChiCTR2100054116). Written informed consent was obtained from all participants.

### Study design and participants

This retrospective–prospective, real-world study included 352 pregnant women with CHB who were enrolled at the infectious disease clinic of the Second Affiliated Hospital of Chongqing Medical University between September 2013 and July 2024. Pregnant women with CHB who had at least one visit during pregnancy or were followed up during the postpartum period between September 2013 and November 2022 were enrolled retrospectively, whereas participants enrolled between November 2022 and July 2024 were included prospectively. The inclusion criteria were hepatitis B surface antigen (HBsAg) positivity, age >18 years, and treatment-naive status. Patients with fatty liver disease, autoimmune liver disease, liver cirrhosis, or coinfection with other hepatitis viruses or human immunodeficiency virus were excluded. Ultimately, 228 HBeAg-positive and 48 HBeAg-negative pregnant women with at least one follow-up visit during pregnancy were included in the analysis. Participants with longitudinal postpartum follow-up included 108 HBeAg-positive pregnant women who received short-term prophylactic antiviral therapy during pregnancy (all postpartum women discontinued antiviral therapy according to current guidelines) and 21 HBeAg-negative pregnant women who did not receive prophylactic antiviral therapy. The flowchart of participant enrollment is shown in **Supplementary Figure S1**.

### Data collection and laboratory testing

Demographic characteristics, including age, parity, gestational week, and time of delivery, as well as treatment regimen and timing of treatment discontinuation, were recorded. Clinical data were also collected, including complete blood count results, liver function tests, alpha-fetoprotein (AFP), hepatitis B surface antigen (HBsAg), hepatitis B e-antigen (HBeAg), and HBV DNA levels, as well as liver stiffness measurements and abdominal ultrasound findings. The neutrophil-to-lymphocyte ratio (NLR) and platelet-to-lymphocyte ratio (PLR) were calculated as surrogate markers of immune status (Zahorec, 2021; Islam et al., 2024). Quantification of HBsAg was performed using the Roche COBAS HBsAg II-Q immunoassay, with a lower limit of detection (LLD) of 0.05 IU/mL. HBeAg quantification was performed using assays from Abbott GmbH & Co. KG, with an LLD of 0.59 PEIU/mL. HBV DNA levels were measured using the Roche Amplicor/COBAS TaqMan HBV Test v2.0 (Roche Molecular Diagnostics, Pleasanton, CA, USA), with an LLD of 100 IU/mL.

### Definitions of natural history phase

Patients were classified according to the natural history phases of HBV infection defined in the 2018 AASLD guidelines (**Supplementary Table S1**). Participants who did not meet the criteria for any of these phases were categorized as being in an indeterminate phase (IP), which was further subdivided into HBeAg-positive and HBeAg-negative IP phases (Terrault et al., 2018). Data were collected during the first, second, and third trimesters of pregnancy. For HBeAg-positive participants, data were collected at 24–28 weeks of gestation (before initiation of antiviral therapy), near the time of delivery, at the end of treatment (EOT), and at routine follow-up visits at 1 month, 3 months, 6 months, and every 6–12 months after EOT. For HBeAg-negative participants, data were collected at 24–28 weeks of gestation, near the time of delivery, and at routine follow-up visits at 3 months, 6 months, and every 6–12 months after delivery. Data collection occurred more frequently in HBeAg-positive participants due to the requirement for close monitoring following short-term antiviral therapy.

### Outcome events

The outcome events included transition to the immune-active (IA) phase, phase maintenance, and liver disease progression to advanced liver fibrosis or cirrhosis. Most participants who transitioned to the IA phase received re-treatment according to the most recent guidelines. Phase maintenance was assessed based on the natural history phase observed at the last two follow-up visits.

Advanced liver fibrosis and cirrhosis were evaluated using non-invasive assessments, including the aspartate aminotransferase (AST)-to-platelet (PLT) ratio index (APRI), the fibrosis index based on four factors (FIB-4), and imaging methods such as transient elastography or abdominal ultrasound. APRI was calculated using the following formula: [AST (U/L)/upper limit of normal (ULN)]/PLT (10^9^/L) × 100, whereas FIB-4 was calculated as follows: [age (years) × AST (U/L)]/[PLT (10^9^/L) × (ALT [U/L])^1/2^] (Xiao et al., 2017) (Liu et al., 2022). Participants who met at least one of the following criteria were considered to have advanced liver fibrosis: (i) APRI ≥1.5; (ii) FIB-4 ≥3.25; or (iii) liver stiffness measurement (LSM) ≥9.0 kPa (ALT <1 × ULN), ≥10.6 kPa (1 × ULN < ALT <2 × ULN), or ≥12.4 kPa (ALT <5 × ULN). Participants who met at least one of the following criteria were considered to have liver cirrhosis: (i) LSM ≥12.0 kPa (ALT <1 × ULN) or ≥17.0 kPa (ALT <5 × ULN); or (ii) abdominal ultrasound findings suggestive of cirrhosis (Consensus on the diagnosis and treatment of hepatic fibrosis (2019), 2020).

### Statistical analysis

Continuous variables were expressed as median (range) and compared using the Mann–Whitney U test. Categorical variables were expressed as frequency (proportion) and compared using the chi-square test or Fisher’s exact test, as appropriate. The cumulative incidence of outcome events was estimated using the Kaplan–Meier method and compared using the log-rank test. The optimal cut-off value for continuous variables was determined using the surv_cutpoint function in the R package survminer. Multivariate Cox proportional hazards regression analysis was used to identify risk factors for phase maintenance and transition to the IA phase. Variables with a p-value <0.05 in univariate Cox regression analyses were entered into the multivariate Cox regression model. The longitudinal dynamics of alanine aminotransferase (ALT), HBV DNA, hepatitis B surface antigen (HBsAg), and HBeAg during follow-up were visualized using color-coded heat maps.

All tests were two sided, and a p-value <0.05 was considered statistically significant. Statistical analyses were performed using SPSS version 24.0 (IBM Corp., Armonk, NY, USA) and R version 4.3.1 (https://www.R-project.org/; IBM Corp, 2016).

## Results

### Distribution and dynamics of natural history phases during pregnancy

The distribution of natural history phases of HBV infection changed from the first to the third trimester of pregnancy. A total of 276 pregnant women with CHB were included in the analysis. In the first trimester, the HBeAg-positive indeterminate phase (IP) was dominant (39.1%), followed by the HBeAg-positive immune-active (IA) phase (24.1%), the immune-tolerant (IT) phase (21.8%), the inactive carrier (IC) phase (8.0%), the HBeAg-negative IP phase (4.6%), and the HBeAg-negative IA phase (2.3%).

**Figure 1:**
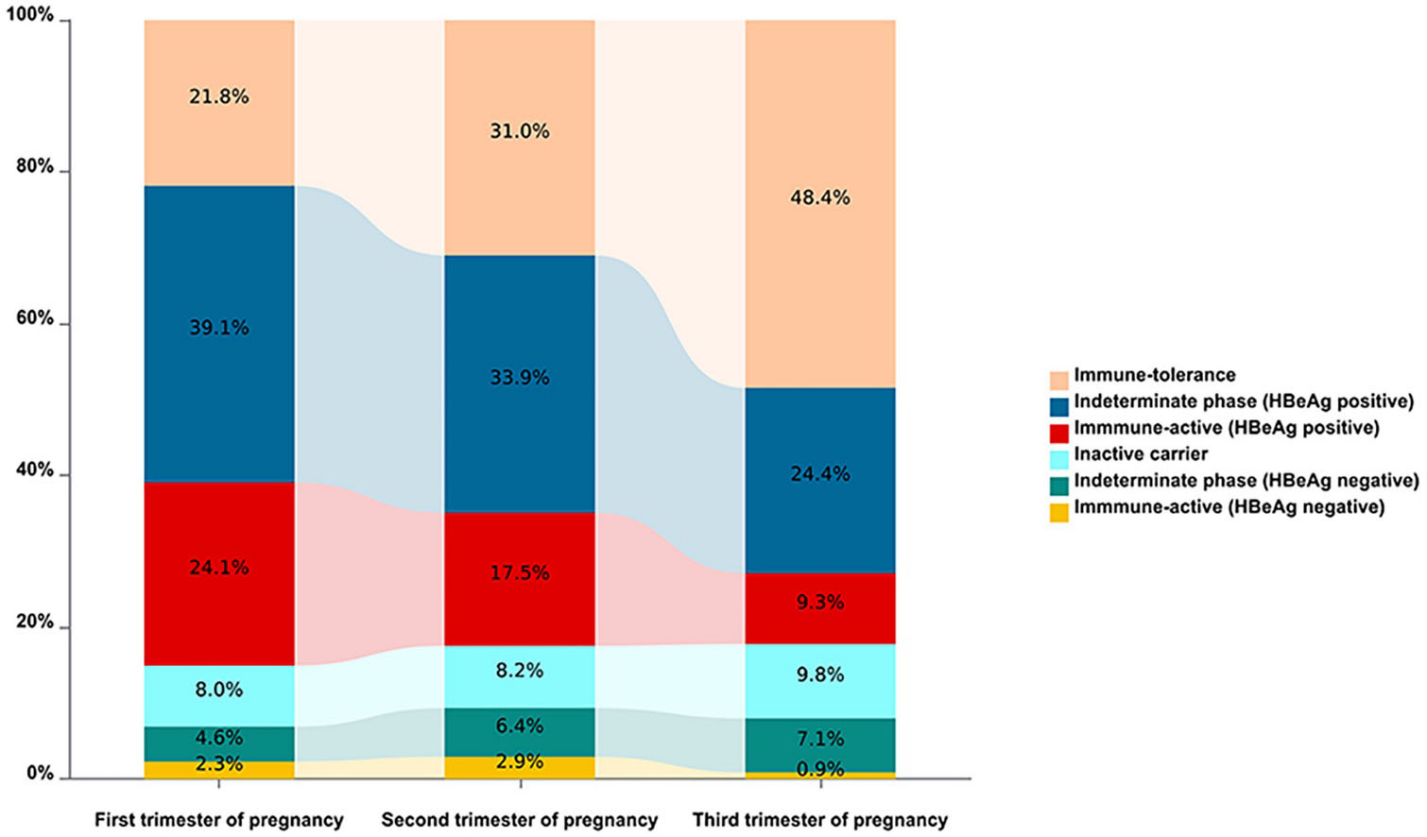
Distribution and natural history dynamics among pregnant women with CHB during pregnancy. Different colors represent different phases. The horizontal axis represents different trimesters of pregnancy, and the vertical axis represents the percentage of each phase. CHB, chronic hepatitis B.

As pregnancy progressed, the proportion of patients in the IT phase increased progressively, whereas the proportions of patients in the HBeAg-positive IP and HBeAg-positive IA phases decreased. The proportions of patients in the IC and HBeAg-negative IP phases remained relatively stable. In the third trimester, most patients were in the IT phase (48.4%), followed by the HBeAg-positive IP phase (24.4%), the IC phase (9.8%), the HBeAg-positive IA phase (9.4%), the HBeAg-negative IP phase (7.1%), and the HBeAg-negative IA phase (0.9%) (**Figure 1**). We further analyzed the dynamics of natural history phases in 41 pregnant women with CHB who had longitudinal follow-up across the first, second, and third trimesters. Consistent with the overall cohort, the proportion of patients in the IT phase gradually increased, whereas that of patients in the HBeAg-positive IA phase gradually decreased as pregnancy progressed (**Supplementary Figure S2**).

### Characteristics of different phases in pregnant women with CHB at the third trimester

The characteristics of pregnant women with CHB in different natural history phases at 24–28 weeks of gestation are summarized in **Supplementary Table S1**. Patients in the IT and IC phases were comparable to those in the IP phase with respect to age at enrollment and at last follow-up, parity, follow-up duration, number of follow-up visits, platelet count, virologic markers, and FIB-4 index, whereas alanine aminotransferase (ALT) levels and the aspartate aminotransferase–to-platelet ratio index (APRI) were lower. The proportion of patients who received tenofovir disoproxil fumarate (TDF) treatment and the duration of treatment were comparable between patients in the IT phase and those in the HBeAg-positive IP phase. Detailed characteristics of participants in each disease phase at 24–28 weeks of gestation are presented in **Supplementary Table S1**.

### Postpartum dynamics of ALT and virologic markers among participants with different phases

Postpartum dynamics of ALT and virologic markers differed across disease phases. Participants in the IT and HBeAg-positive IP phases exhibited a higher proportion of abnormal ALT levels and commonly experienced viral rebound during the postpartum period, whereas participants in the IC and HBeAg-negative phases maintained normal ALT levels and relatively stable virologic markers.

**Figure 2:**
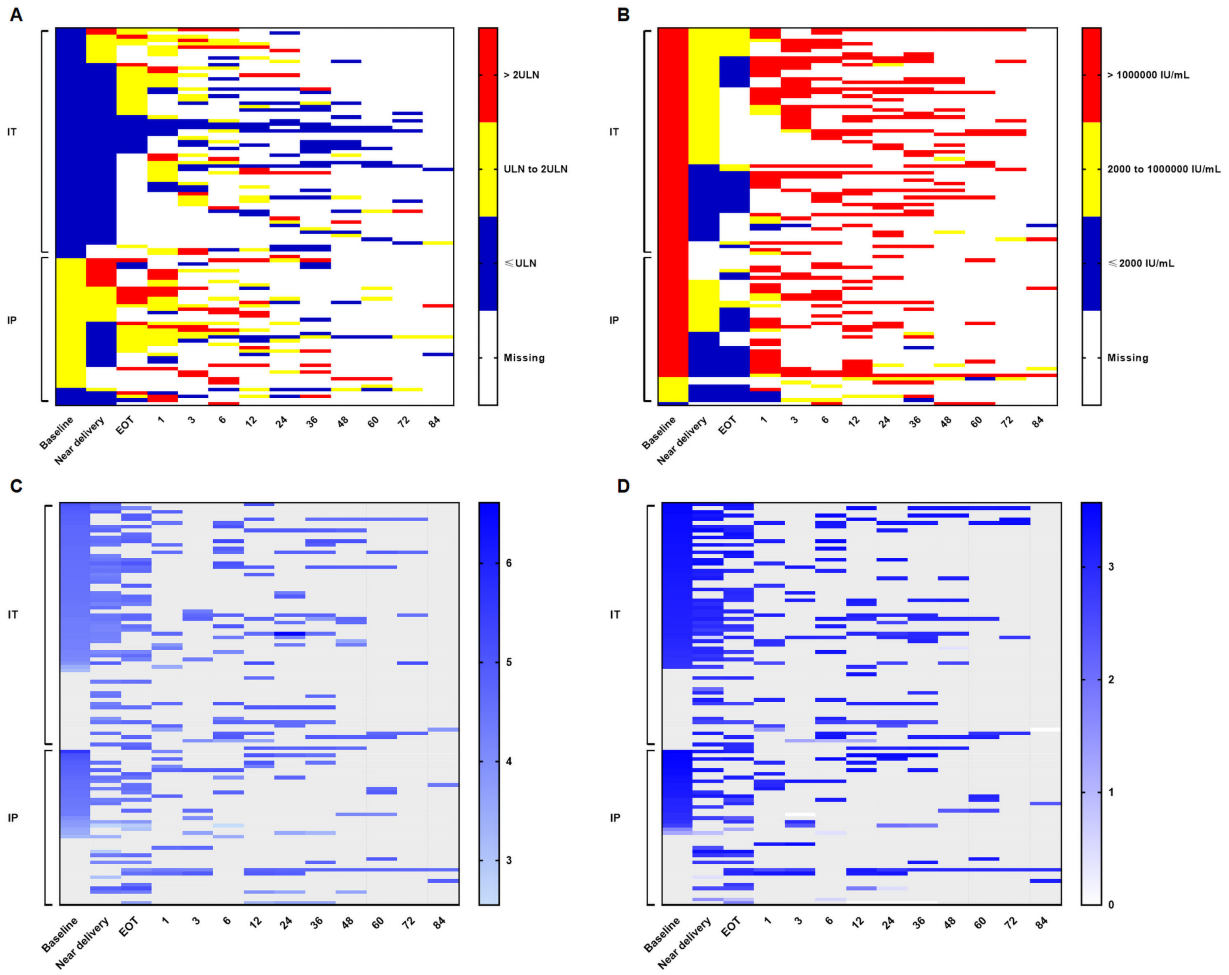
Dynamics of ALT and virologic markers in the IT and HBeAg-positive IP phases. Color-coded heat maps depict the dynamics of ALT and virologic markers during pregnancy and the postpartum period for each HBeAg-positive participant. The horizontal axis represents time points, and numbers indicate months after treatment discontinuation. The vertical axis represents individual participants stratified by IT and HBeAg-positive IP. **(A)** Blue, yellow, and red tiles indicate ALT ≤ 1ULN, ALT 1ULN to 2 ULN, and ALT > 2ULN, respectively. White tiles indicate missing data. The ULN for female patients was 25 U/L. **(B)** Blue, yellow, and red tiles indicate HBV DNA ≤ 2,000 IU/mL, HBV DNA 2,000 to 1,000,000 IU/mL, and HBV DNA > 1,000,000 IU/mL, respectively. White tiles indicate missing data. **(C, D)** Panels show HBsAg and HBeAg levels, respectively, with higher levels indicated by darker tile colors. Gray and white tiles represent missing data and HBeAg clearance, respectively. Spontaneous HBeAg clearance occurred after treatment discontinuation in one participant in the IT phase and two participants in the IP phase. ALT, alanine aminotransferase; DNA, deoxyribonucleic acid; EOT, end of treatment; HBeAg, hepatitis B e antigen; HBsAg, hepatitis B surface antigen; HBV, hepatitis B virus; IP, indeterminate phase; IT, immune tolerance; ULN, upper limit of normal.

The median postpartum follow-up time for participants in the IT and HBeAg-positive IP phases was 31.5 months (0.9–81.8 months) and 16.1 months (0.2–95.7 months), respectively. Color-coded heat maps (**Figure 2A**) show that most participants in the IT phase maintained normal ALT levels at delivery, with a small proportion exhibiting ALT levels between 1 × ULN and 2 × ULN. Following treatment discontinuation within 3 months postpartum, the majority of participants displayed ALT levels between 1 × ULN and 2 × ULN. During long-term postpartum follow-up, ALT levels returned to the normal range in most participants.

In contrast, ALT dynamics in participants in the HBeAg-positive IP phase differed markedly from those in the IT phase. Abnormal ALT levels (>1 × ULN) were observed in most participants near delivery, with a high proportion exhibiting ALT levels exceeding 2 × ULN during the early postpartum period after treatment discontinuation and throughout long-term postpartum follow-up (**Figure 2A**). Consistently, ALT levels in participants in the HBeAg-positive IP phase were significantly higher than those in participants in the IT phase at corresponding time points (**Supplementary Figure S3**). With respect to HBV DNA dynamics, the proportion of participants in the IT phase with HBV DNA levels below 2000 IU/mL near delivery was lower than that in participants in the HBeAg-positive IP phase. In both groups, HBV DNA levels rebounded rapidly to >1,000,000 IU/mL during the early postpartum period following treatment discontinuation and remained at high levels during long-term postpartum follow-up (**Figure 2B**). In addition, hepatitis B surface antigen (HBsAg) and HBeAg levels remained relatively stable during pregnancy and throughout the postpartum follow-up period in both groups (**Figures 2C, D**).

**Figure 3:**
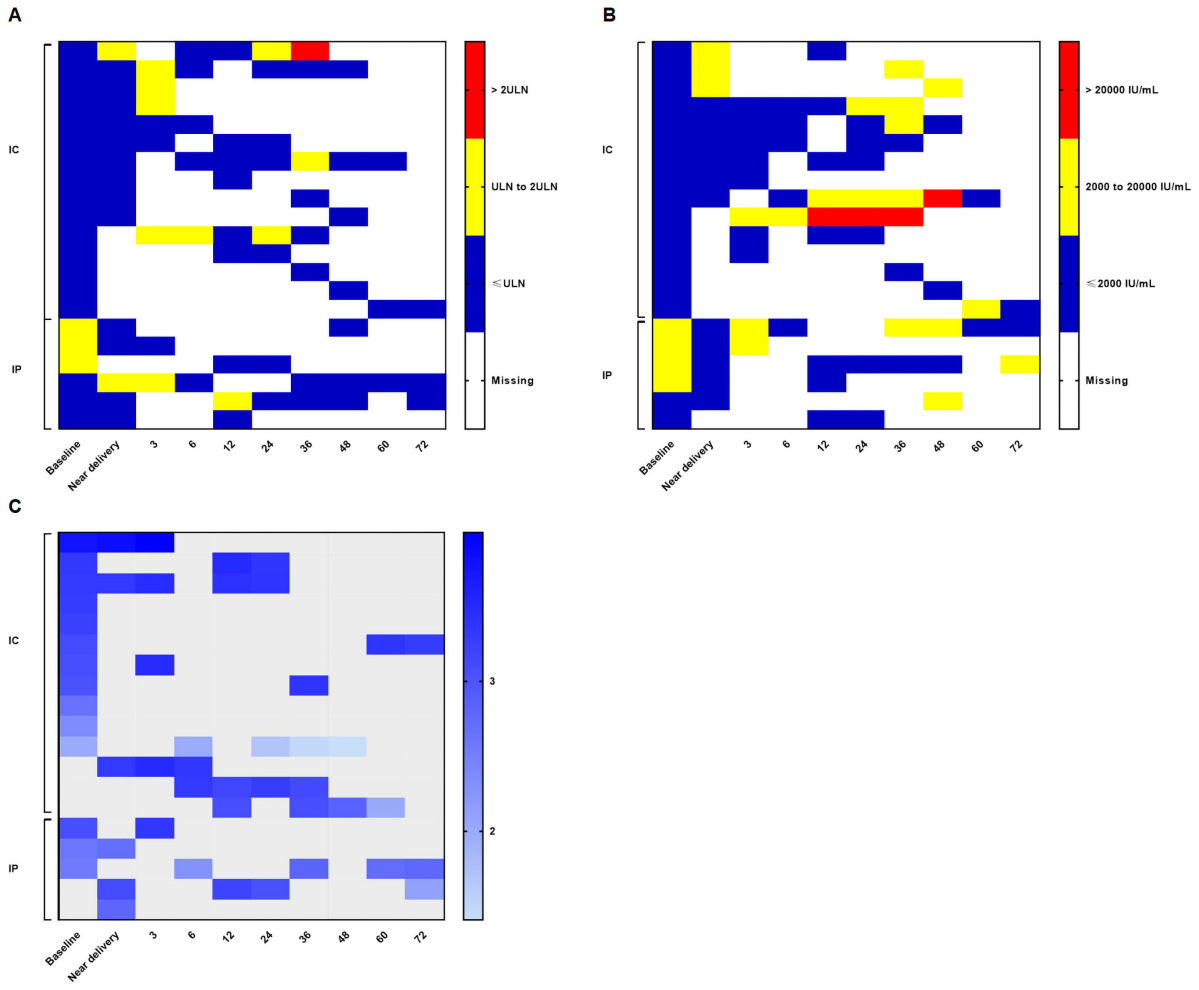
Dynamics of ALT and virologic markers in the IT and HBeAg-negative IP phases. Color-coded heat maps depict the dynamics of ALT and virologic markers during pregnancy and the postpartum period for each HBeAg-negative participant. The horizontal axis represents time points, with numbers indicating months after delivery. The vertical axis represents individual participants stratified by IC and HBeAg-negative IP. **(A)** Blue, yellow, and red tiles indicate ALT ≤ 1ULN, ALT 1ULN to 2 ULN, and ALT > 2ULN, respectively. White tiles indicate missing data. The ULN for female patients was 25 U/L. **(B)** Blue, yellow, and red tiles indicate HBV DNA ≤ 2,000 IU/mL, HBV DNA 2,000 to 1,000,000 IU/mL, and HBV DNA > 1,000,000 IU/mL, respectively. White tiles indicate missing data. **(C)** Panel shows HBsAg levels, with higher levels indicated by darker tile colors. Gray tiles indicate missing data. ALT, alanine aminotransaminase; DNA, deoxyribonucleic acid; HBeAg, hepatitis B e antigen; HBsAg, hepatitis B surface antigen; HBV, hepatitis B virus; IP, indeterminate phase; IT, immune-tolerance. ULN, upper limit of normal.

The median postpartum follow-up time for participants in the IC and HBeAg-negative IP phases was 32.0 months (0.9–56.9 months) and 34.5 months (1.3–75.1 months), respectively. Color-coded heat maps (**Figure 3**) indicate that ALT levels remained within the normal range in most participants in both groups after the early postpartum period, with ALT levels exceeding 2 × ULN observed in only a few participants. HBV DNA and HBsAg levels also remained relatively stable during pregnancy and throughout the postpartum follow-up period.

### Cumulative incidence and predictors of phase transition to IA among participants with different phases

The cumulative incidence of phase transition to the immune-active (IA) phase was higher among participants in the immune-tolerant (IT) and HBeAg-positive indeterminate phase (IP) groups and lower among participants in the inactive carrier (IC) and HBeAg-negative IP groups. Overall, 31.8% (21/66) of participants in the IT phase, 71.4% (30/42) of those in the HBeAg-positive IP phase, 6.7% (1/15) of those in the IC phase, and none (0/6) of those in the HBeAg-negative IP phase experienced phase transition to IA. Details of postpartum phase transitions are shown in **Supplementary Figure S4**.

**Figure 4:**
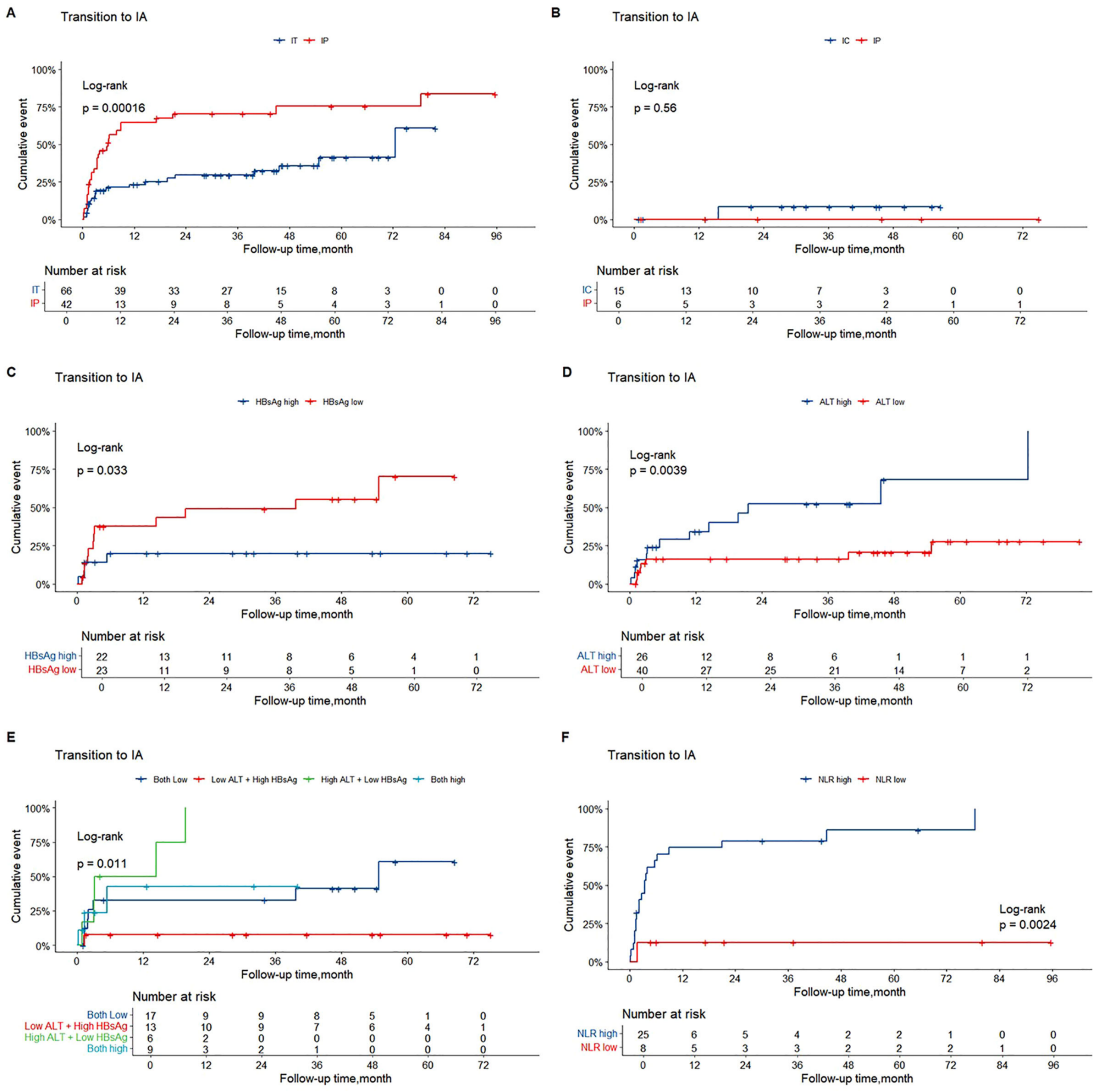
Cumulative incidences of transition to IA phase among participants in different phases. The horizontal axis represents postpartum follow-up after treatment discontinuation, and the vertical axis represents cumulative incidence. **(A)** Cumulative incidence of transition to the HBeAg-positive IA phase among participants in the IT and HBeAg-positive IP phases. **(B)** Cumulative incidence of transition to the HBeAg-negative IA phase among participants in the IT and HBeAg-negative IP phases. **(C, D)** Cumulative incidence of transition to the HBeAg-positive IA phase among participants in the IT phase stratified by HBsAg and ALT levels. **(E)** Cumulative incidence of transition to the HBeAg-positive IA phase among participants in the IT phase stratified by combined HBsAg and ALT levels. **(F)** Cumulative incidence of transition to the IA phase among participants in the HBeAg-positive IP phase stratified by neutrophil-to-lymphocyte ratio (NLR). ALT, alanine aminotransaminase; HBsAg, hepatitis B surface antigen; IA, immune-active; IC, inactive carrier; IP, indeterminate phase; IT, immune-tolerance; LdT, telbivudine; NLR, neutrophil to lymphocyte ratio; TDF, tenofovir disoproxil fumarate.

The cumulative incidence of transition to the HBeAg-positive IA phase was significantly higher among participants in the HBeAg-positive IP phase than among those in the IT phase (*p* = 0.00016). By contrast, the cumulative incidence of transition to the IA phase was comparable between participants in the IC and HBeAg-negative IP phases (*p* = 0.320; **Figures 4A, B**).

Due to the limited number of outcome events, predictors of phase transition to IA were analyzed only in participants in the IT and HBeAg-positive IP phases. Multivariate Cox regression analysis in participants in the IT phase indicated that a lower baseline level of hepatitis B surface antigen (HBsAg) (hazard ratio [HR], 0.163; 95% confidence interval [CI], 0.035–0.771; *p* = 0.022) and a higher baseline level of alanine aminotransferase (ALT) (HR, 1.146; 95% CI, 1.011–1.299; *p* = 0.033) were independent predictors of phase transition to IA (**Table 1**). The optimal cutoff values for HBsAg and ALT were 4.53 log_10_ IU/mL and 17 U/L, respectively. The cumulative incidence of phase transition to IA was significantly higher among participants with lower HBsAg levels and higher ALT levels than among those with higher HBsAg levels and lower ALT levels (**Figures 4C–E**).

**Table 1:** Cox regression analysis of risk factors associated with transition to the IA phase among pregnant women in the IT and HBeAg-positive IP phases.

Variable	Univariate				Multivariate		
		HR	95% CI	p-value	HR	95% CI	p*-*value
IT phase							
Age		0.967	0.874-1.070	0.512			
Treatment time, month		0.782	0.535-1.142	0.203			
First pregnancy	Yes	1.508	0.504-4.513	0.463			
	No	Reference					
Treatment regimen	TDF	0.924	0.371-2.304	0.866			
	LdT	Reference					
ALT, U/L		1.097	1.004-1.198	0.041	1.146	1.011-1.299	0.033
HBsAg, log IU/mL		0.183	0.042-0.805	0.025	0.163	0.035-0.771	0.022
HBeAg, log PEIU/mL		0.422	0.556-7.441	0.556			
HBV DNA, log IU/mL		1.030	0.422-2.516	0.948			
APRI		2.678	0.074-96.542	0.590			
FIB4		0.540	0.100-2.917	0.474			
NLR		0.918	0.667-1.262	0.598			
PLR		1.000	0.987-1.012	0.975			
HBeAg-positive IP phase							
Age		1.128	1.013-1.255	0.028			
Treatment time, month		1.075	0.883-1.310	0.471			
First pregnancy	Yes	0.595	0.250-1.415	0.240			
	No	Reference					
Treatment regimen	TDF	2.859	1.179-6.932	0.020			
	LdT	Reference					
ALT, U/L		0.986	0.936-1.040	0.613			
HBsAg, log IU/mL		0.645	0.259-1.606	0.346			
HBeAg, log PEIU/mL		0.691	0.329-1.448	0.327			
HBV DNA, log IU/mL		0.943	0.648-1.372	0.758			
APRI		5.882	0.415-83.314	0.190			
FIB-4		7.435	1.556-35.540	0.012			
NLR		1.583	1.159-2.161	0.004	1.564	1.146-2.133	0.005
PLR		0.996	0.982-1.009	0.518			

Variables with an associated p-value < 0.05 were included in the multivariate Cox regression analysis.

ALT, alanine aminotransaminase; APRI, aspartate aminotransferase to platelet ratio index; CI, confidence interval; DNA, deoxyribonucleic acid; FIB-4, fibrosis index based on four factors; HBeAg, hepatitis B e antigen; HBsAg, hepatitis B surface antigen; HBV, hepatitis B virus; HR, hazard ratio; IT, immune-tolerance; IP, indeterminate phase; LdT, telbivudine; NLR, neutrophil to lymphocyte ratio; PLR, platelet to lymphocyte ratio; TDF, tenofovir disoproxil fumarate.

Multivariate Cox regression analysis in participants in the HBeAg-positive IP phase identified a higher baseline neutrophil-to-lymphocyte ratio (NLR) as an independent predictor of transition to the IA phase (HR, 1.564; 95% CI, 1.146–2.133; p = 0.005) (**Table 1**). The optimal cutoff value for NLR was 3.32, and the cumulative incidence of phase transition to IA was significantly higher among participants with higher NLR levels than among those with lower NLR levels (**Figure 4F**).

### Cumulative incidence and predictors of phase maintenance among participants with different phases

The cumulative incidence of phase maintenance was higher among participants in the IC and HBeAg-negative IP phases and lower among participants in the IT and HBeAg-positive IP phases. Overall, 51.5% (34/66) of participants in the IT phase maintained their disease phase after postpartum treatment discontinuation, which was significantly higher than the 28.6% (12/42) observed among participants in the HBeAg-positive IP phase (*p* = 0.028). In addition, 66.7% (10/15) of participants in the IC phase maintained their phase, and an identical proportion was observed among participants in the HBeAg-negative IP phase (66.7%; 4/6; *p* = 1.000).

**Figure 5:**
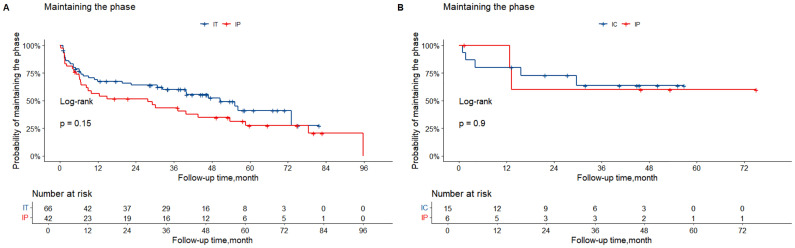
Cumulative incidences of phase maintenance among participants in different phases. The horizontal axis represents postpartum follow-up after treatment discontinuation, and the vertical axis represents cumulative incidence. **(A)** Cumulative incidence of phase maintenance among HBeAg-positive pregnant women. Red and blue lines represent participants in the HBeAg-positive IP and IT phases, respectively. **(B)** Cumulative incidence of phase maintenance among HBeAg-negative pregnant women. Red and blue lines represent participants in the HBeAg-negative IP and IC phases, respectively. IA, immune-active; IC, inactive carrier; IP, indeterminate phase; IT, immune-tolerance; LdT, telbivudine; TDF, tenofovir disoproxil fumarate.

As shown in **Figure 5A**, the cumulative incidence of phase maintenance was higher among participants in the IT phase than among those in the HBeAg-positive IP phase, although the difference was not statistically significant (*p* = 0.140). The cumulative incidence of phase maintenance was comparable between participants in the IC and HBeAg-negative IP phases (*p* = 0.560; **Figure 5B**).

Given the limited number of outcome events, predictors of phase maintenance were analyzed only in participants in the IT and HBeAg-positive IP phases. Multivariate Cox regression analysis showed that a higher baseline HBV DNA level (HR, 2.984; 95% CI, 1.349–6.603; *p* = 0.007) and tenofovir disoproxil fumarate (TDF) treatment during pregnancy (HR, 2.473; 95% CI, 1.147–5.335; *p* = 0.021) were independent predictors of phase maintenance in the IT phase (**Table 2**). No independent predictors of phase maintenance were identified for participants in the HBeAg-positive IP phase.

**Table 2:** Cox regression analysis of risk factors associated with maintenance in the IT phase.

Variable	Univariate				Multivariate		
		HR	95% CI	p-value	HR	95% CI	p-value
Age		1.032	0.955-1.115	0.430			
Treatment time, month		1.339	0.996-1.799	0.053			
First pregnancy	Yes	0.797	0.368-1.726	0.566			
	No	Reference					
Treatment regimen	TDF	2.874	1.317-6.275	0.008	2.984	1.349-6.603	0.007
	LdT	Reference					
ALT, U/L		1.033	0.951-1.121	0.445			
HBsAg, log IU/mL		1.409	0.247-8.055	0.700			
HBeAg, log PEIU/mL		0.191	0.016-2.264	0.190			
HBV DNA, log IU/mL		2.062	1.040-4.088	0.038	2.473	1.147-5.335	0.021
APRI		2.412	0.030-195.986	0.695			
FIB-4		1.373	0.417-4.516	0.602			
NLR		1.125	0.892-1.419	0.318			
PLR		1.005	0.995-1.015	0.344			

Variables with an associated p-value < 0.05 were included in the multivariate Cox regression analysis.

ALT, alanine aminotransaminase; APRI, aspartate aminotransferase to platelet ratio index; CI, confidence interval; DNA, deoxyribonucleic acid; FIB-4, fibrosis index based on four factors; HBeAg, hepatitis B e antigen; HBsAg, hepatitis B surface antigen; HBV, hepatitis B virus; HR, hazard ratio; IT, immune-tolerance; LdT, telbivudine; NLR, neutrophil to lymphocyte ratio; PLR, platelet to lymphocyte ratio; TDF, tenofovir disoproxil fumarate.

### Risk of liver disease progression in different phases

**Figure 6:**
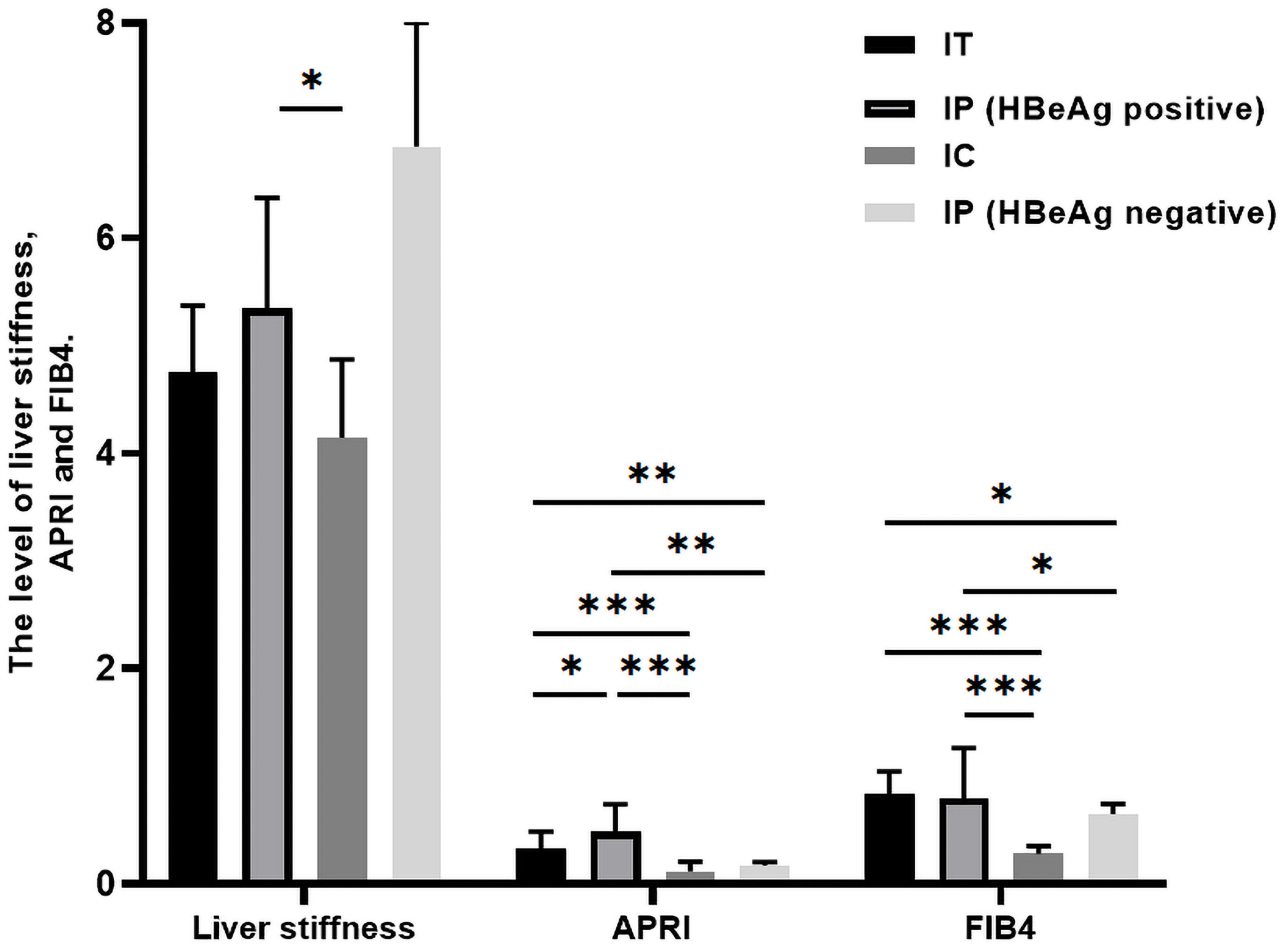
Liver disease progression evaluated by non-invasive tests according to disease phase. The horizontal axis represents non-invasive tests (liver stiffness, APRI, and FIB-4), and the vertical axis represents test values. Colored bars represent participants in different disease phases. p-values <0.05, <0.01, and <0.001 are indicated by *, **, and ***, respectively. APRI, aspartate aminotransferase–to-platelet ratio index; FIB-4, fibrosis index based on four factors; HBeAg, hepatitis B e antigen; IA, immune-active; IC, inactive carrier; IP, indeterminate phase; IT, immune-tolerance.

The risk of postpartum liver disease progression was low among pregnant women across all phases. Only three participants (all in the immune-tolerant [IT] phase) progressed to advanced liver fibrosis, and no cases of cirrhosis or hepatocellular carcinoma were observed. We compared non-invasive liver fibrosis indices, including liver stiffness, the aspartate aminotransferase–to-platelet ratio index (APRI), and the fibrosis index based on four factors (FIB-4), among patients in different phases and found significantly higher liver stiffness values in participants in the HBeAg-positive indeterminate phase (IP) compared with those in the inactive carrier (IC) phase. In addition, APRI and FIB-4 values remained within the normal range during long-term follow-up but were significantly higher in participants in the IT and HBeAg-positive IP phases than in those in the IC and HBeAg-negative IP phases. No significant differences were observed in any non-invasive fibrosis indices between participants in the IT and IC phases and those in the IP phase, except for higher APRI values in participants in the HBeAg-positive IP phase (**Figure 6**).

## Discussion

This study provides, to our knowledge, the first longitudinal description of HBV natural history phase dynamics during pregnancy and the postpartum period in women with chronic hepatitis B (CHB). The main finding of this study is that pregnancy exerts a differential effect on the natural history of HBV infection. We observed a dominant and progressively increasing prevalence of the immune-tolerant (IT) phase as pregnancy advanced. HBeAg-positive women, particularly those in the indeterminate phase (IP) at 24–28 weeks of gestation, demonstrated a significantly increased risk of transitioning to the immune-active (IA) phase after delivery. In contrast, HBeAg-negative women, including those in the inactive carrier (IC) and HBeAg-negative IP phases, exhibited notable phase stability. Reassuringly, the short-term risk of liver disease progression was low across all phases. These findings provide insight into pregnancy-associated dynamics of HBV infection and may help inform the management of this specific population.

Although the natural history of HBV infection is well characterized in the general CHB population, data specific to pregnant women with CHB remain limited. Previous observations have shown that alanine aminotransferase (ALT) levels tend to decrease across trimesters during pregnancy, potentially reflecting modulation of hepatic inflammation by gestational immune suppression (Giles et al., 2015), which is consistent with our finding of a progressively increasing prevalence of the IT phase throughout pregnancy. Our results suggest a divergence in pregnancy-associated phase dynamics according to HBeAg status. This difference may be attributable to distinct immune profiles associated with HBeAg positivity or negativity, as suggested by prior researches (Joshi et al., 2017; Li et al., 2019). Furthermore, our previous work demonstrating differential levels of novel virological markers, such as pregenomic RNA and hepatitis B core-related antigen, between HBeAg-positive and HBeAg-negative pregnant women may partially explain these observations (Wang et al., 2022). Nevertheless, pregnancy-related factors, including hormonal fluctuations and systemic immunomodulation, are known to influence HBV replication, and their differential effects according to HBeAg status warrant further investigation. Larger-scale longitudinal studies are needed to elucidate the relative contributions of these mechanisms.

The postpartum period represents a critical window during which the reversal of gestational immune tolerance may alter the course of HBV infection. This shift may manifest as a postpartum cytokine flare, potentially triggering alanine aminotransferase (ALT) flares, viral rebound, or HBeAg clearance (Lin et al., 2006; Joshi et al., 2017; Samadi Kochaksaraei et al., 2020; Wang et al., 2021; Wang et al., 2022; Tang et al., 2024; Tang et al., 2025). Our previous research in HBeAg-positive pregnant women substantiates this link, demonstrating a close association between specific cytokine profiles and virologic markers, thereby highlighting the dynamic interaction between immunity and HBV replication postpartum (Tang et al., 2024). The natural history phase, a composite index of host immunity, viral replication (HBV DNA level and HBeAg status), and liver inflammation (ALT), is consequently susceptible to these postpartum changes. In the present study, our findings suggest that delivery may exert a more discernible impact on the natural history of HBeAg-positive women than on that of HBeAg-negative patients, a pattern consistent with the higher reported incidence of postpartum ALT flares in HBeAg-positive individuals (OuYang et al., 2024). Notably, a substantial proportion of participants remained at risk of transition to the immune-active (IA) phase 6 months after postpartum treatment discontinuation, particularly those in the HBeAg-positive indeterminate phase (IP). Given that the early postpartum period (within 3 months after delivery) is recognized as a critical window for virologic, hepatic inflammatory, and immune alterations (OuYang et al., 2024; Wang et al., 2025), our findings underscore the necessity for extended and vigilant monitoring of HBeAg-positive patients, especially those in the IP phase, to ensure timely identification and management of individuals requiring re-initiation of antiviral therapy.

Our study reveals a notably low risk of postpartum liver disease progression across all phases of HBV infection in pregnant women, in contrast to the established risk profile observed in the general CHB population. In non-pregnant patients with CHB, the IP phase is recognized as a higher-risk state for liver disease progression, with a recent meta-analysis reporting an annual cirrhosis incidence of 0.67% in this population (Huang et al., 2022; Wang et al., 2022; Che-To Lai et al., 2024). In our cohort, however, pregnant women in both the immune-tolerant (IT) and inactive carrier (IC) phases exhibited a similarly low risk of liver disease progression compared with those in the IP phase. This attenuated risk profile may be explained by several factors specific to our study population. All participants were young women, and female sex is a known protective factor against liver disease progression in CHB (Ndow et al., 2024). Moreover, postpartum patients who experienced phase transition to IA received close monitoring and timely antiviral therapy. Collectively, these factors, including female sex, young age, and proactive clinical management, may account for the low observed risk of liver disease progression. Nevertheless, large-scale, long-term prospective studies are needed to further clarify postpartum liver disease progression among pregnant women across different phases of HBV infection.

This study provides the first longitudinal characterization of HBV natural history dynamics throughout pregnancy and the postpartum period and assesses the associated risk of liver disease progression stratified by natural history phase, thereby providing important evidence to inform the management of pregnant women with CHB. However, several limitations should be acknowledged. First, this was a retrospective–prospective study, and not all participants were followed longitudinally throughout pregnancy, which may have introduced selection bias. Second, although reasonable given the relative rarity of CHB in pregnancy, the sample size may limit the generalizability of the findings and the statistical power for subgroup analyses. Third, only the AASLD criteria were used for natural history phase classification. Finally, not all participants underwent repeated testing of all relevant indicators at each phase assessment time point, which may have affected the precision of phase classification. Future multicenter, large-scale, long-term prospective studies are warranted to validate these findings and to elucidate the underlying immunological and virological mechanisms driving the observed phase dynamics.

## Conclusions

In conclusion, our study demonstrates that pregnancy differentially alters the natural history of HBV infection, with a pronounced impact on HBeAg-positive women, particularly those in the indeterminate phase at 24–28 weeks of gestation. This subgroup represents a higher-risk population that warrants intensified and prolonged postpartum monitoring to ensure timely identification of, and antiviral therapy initiation for, individuals progressing to the immune-active phase.

## Data Availability

The raw data supporting the conclusions of this article will be made available by the authors, without undue reservation.
