# Supplementary material for: Higher risk of postpartum phase transition to immune-active among HBeAg-positive pregnant women with indeterminate phase
Source: Front Cell Infect Microbiol. 2026 Jan 7;15:1652690. doi: 10.3389/fcimb.2025.1652690 (PMC12819656; doi:10.3389/fcimb.2025.1652690)
Supplement: Supplementary file 1 [file DataSheet1.docx]

**Higher risk of postpartum phase transition to immune-active among HBeAg-positive pregnant women with indeterminate phase**

Qiao Tang^1*^, Chunrui Wang^1^, Hu Li^1^, Zhiwei Chen^1^, Xiaoqing Liu^1^, Yunling Xue^1^, Yue Qiu^1^, Nan Cai^1^, Yi Zeng^1^, Peng Hu^1, 2#^

**Supplementary tables**

**Table S1 The natural history phases defined by American Association for the Study of Liver Diseases 2018 criteria by practice setting**

| Phases | HBeAg | HBV DNA, IU/mL | ALT, U/L | Liver inflammation and fibrosis |
| --- | --- | --- | --- | --- |
| Immune-tolerance | Positive | > 1 million | < 1 × ULN * | Minimal inflammation, no fibrosis |
| Immune-Active | Positive | ≥ 20,000 | ≥ 2 × ULN | Moderate or severe inflammation with or without fibrosis |
|  | Negative | ≥ 2000 |  |  |
| Inactive carrier | Negative | < 2000 | < 1 × ULN | Absence of significant inflammation |
| Indeterminate phase | Positive | Any HBeAg-positive patient who dose not fit the criteria | | |
|  | Negative | Any HBeAg-negative patient who dose not fit the criteria | | |

* The ALT ULN levels were 25 U/L for females.

Abbreviations: ALT, alanine aminotransaminase; DNA, deoxyribonucleic acid; HBeAg, hepatitis B e antigen; HBV, hepatitis B virus; ULN, upper limit of normal.

**Table S2 The baseline characteristics among pregnant women with different phases**

| Variables | HBeAg positive | | HBeAg negative | |
| --- | --- | --- | --- | --- |
|  | Immune-tolerance (n=66) | Indeterminate phase (n=42) | Inactive carrier (n=15) | Indeterminate phase (n=6) |
| Age at enrollment, year | 28.0 (19.8 - 38.3) | 29.1 (21.7 - 38.2) | 29.2 (27.3 - 45.8) | 30.2 (26.2 - 35.0) |
| Age at last follow-up, year | 30.7 (23.2 - 42.4) | 31.4 (22.7 - 45.6) | 32.1 (29.1 - 48.3) | 34.4 (27.9 - 36.4) |
| First pregnancy, n (%) | 48 (72.7%) | 33 (78.6%) | 8 (53.3%) | 4 (66.7%) |
| TDF treatment, n (%) | 31 (47.0%) | 24 (57.1%) | NA | NA |
| Treatment time, month | 4.4 (1.8 - 9.2) | 3.9 (1.0 - 10.9) | NA | NA |
| Follow-up time, month | 31.5 (0.9 - 81.8) | 16.1 (0.2 - 95.7) | 32.0 (0.9 - 56.9) | 34.5 (1.3 - 75.1) |
| Platelet, 10^9^/L | 185 (112 - 330) | 185.5 (87 - 361) | 211 (67 - 247) | 148 (90 - 184) |
| ALT, U/L | 16 (4 - 26) ^***^ | 32 (8 - 49) | 16 (6 - 24) ^#^ | 25 (13 - 30) |
| HBsAg, log IU/mL | 4.5 (3.4 - 5.1) | 4.5 (3.2 - 5.6) | 3.08 (2.00 - 3.75) | 2.60 (2.55 - 3.06) |
| HBeAg, log PEIU/mL | 3.2 (2.9 - 3.5) | 3.2 (1.2 - 3.6) | NA | NA |
| HBV-DNA, log IU/mL | 7.1 (6.0 - 8.2) | 7.3 (4.3 - 9.0) | 2.00 (0.00 - 3.15) | 3.62 (0.00 - 3.99) |
| APRI | 0.3 (0.1 - 0.8) ^***^ | 0.4 (0.2 - 0.8) | 0.26 (0.18 - 0.71) ^#^ | 0.38 (0.24 - 0.47) |
| FIB-4 | 0.8 (0.4 - 1.7) | 0.8 (0.3 - 1.5) | 0.73 (0.54 - 2.07) | 0.81 (0.48 - 1.50) |
| NLR | 4.3 (2.2 - 8.0) | 4.1 (2.1 - 7.1) | 4.3 (3.0 - 5.5) | 4.2 (3.5 - 7.3) |
| PLR | 121.9 (68.6 - 224.7) | 109.9 (68.5 - 280.0) | 111.1 (53.3 - 196.6) | 90.2 (64.8 - 156.1) |

Note: The statistically significant difference between IT and indeterminate phases (HBeAg positive) were marked by * (*p* < 0.05) and *** (*p* < 0.001). The statistically significant difference between IC and indeterminate phases (HBeAg negative) were marked by # (*p* < 0.05). Abbreviations: ALT, alanine aminotransaminase; APRI, aspartate aminotransferase to platelet ratio index; DNA, deoxyribonucleic acid; FIB-4, fibrosis index based on four factors; HBeAg, hepatitis B e antigen; HBsAg, hepatitis B surface antigen; HBV, hepatitis B virus;NLR, neutrophil to lymphocyte ratio; PLR, platelet to lymphocyte ratio; TDF, tenofovir disoproxil fumarate.

**Supplementary figures**

**
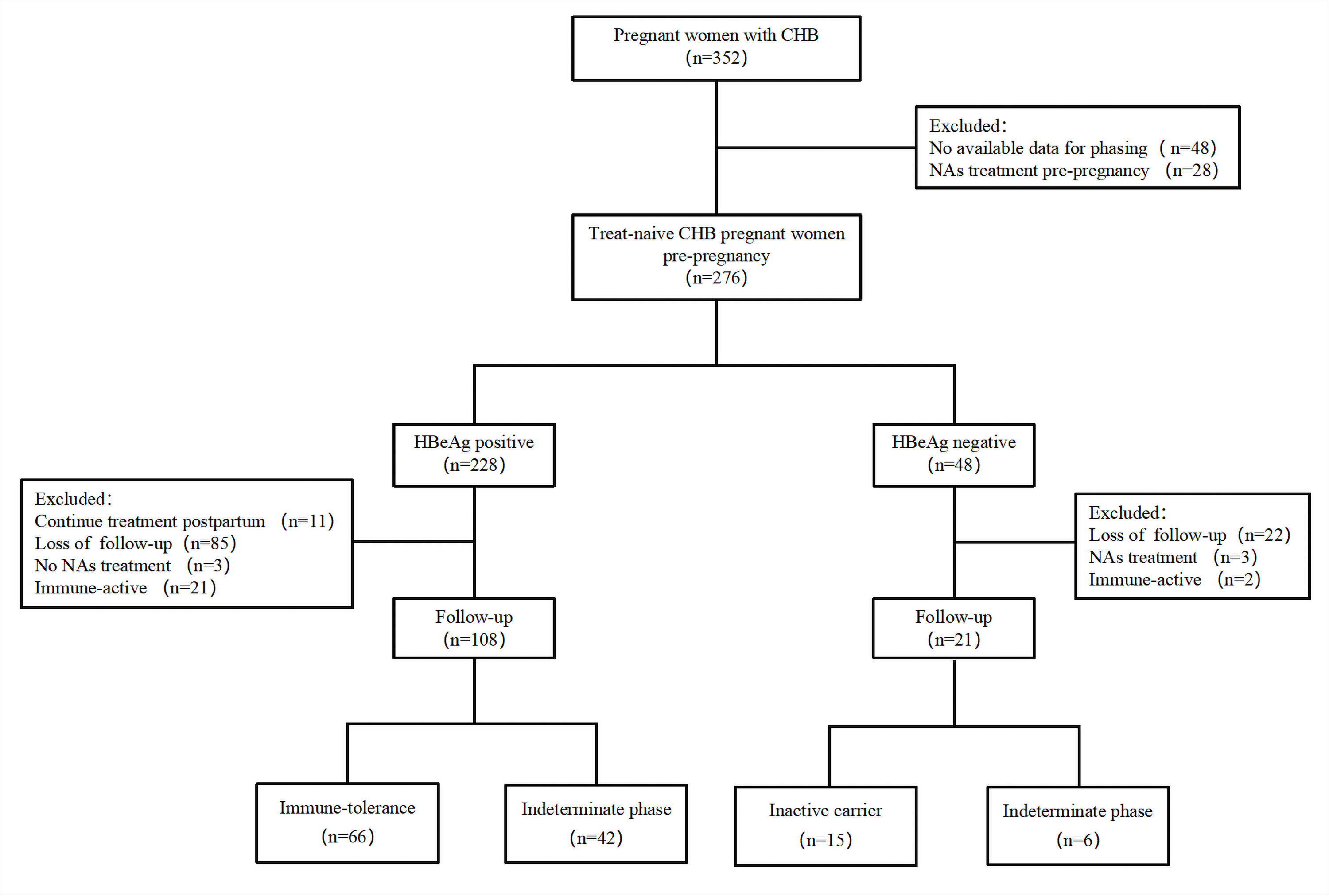
**

**Fig. S1 The flowchart of study participants.** NAs, nucleoside analogues; CHB, chronic hepatitis B; HBeAg, hepatitis B e antigen.


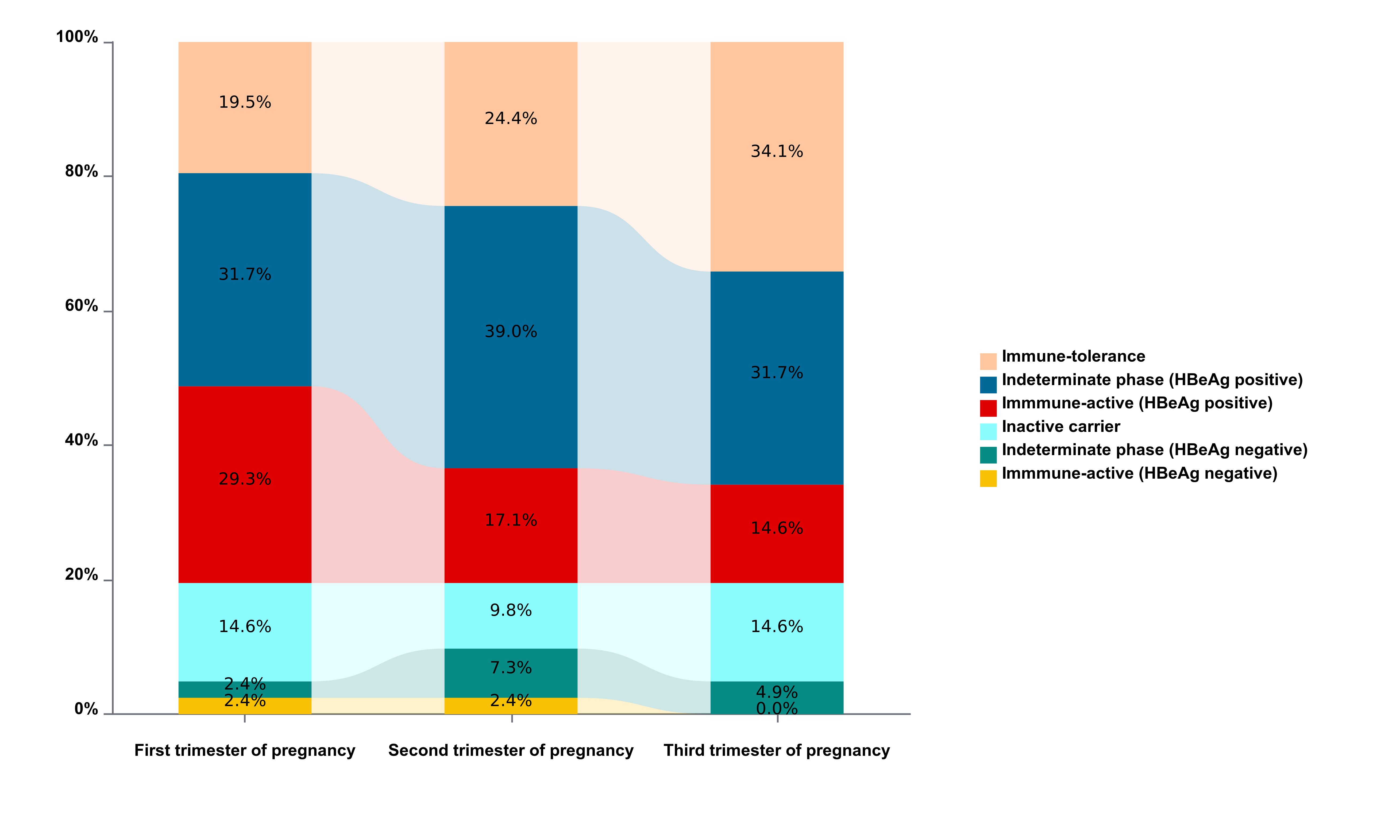


**Fig. S2 The distribution and dynamic of natural history among participants who simultaneously had follow-up in three trimesters.** Different colors represent different phases. The horizontal axis represents different trimester of pregnancy and the vertical axis represents the percentages of different phases.


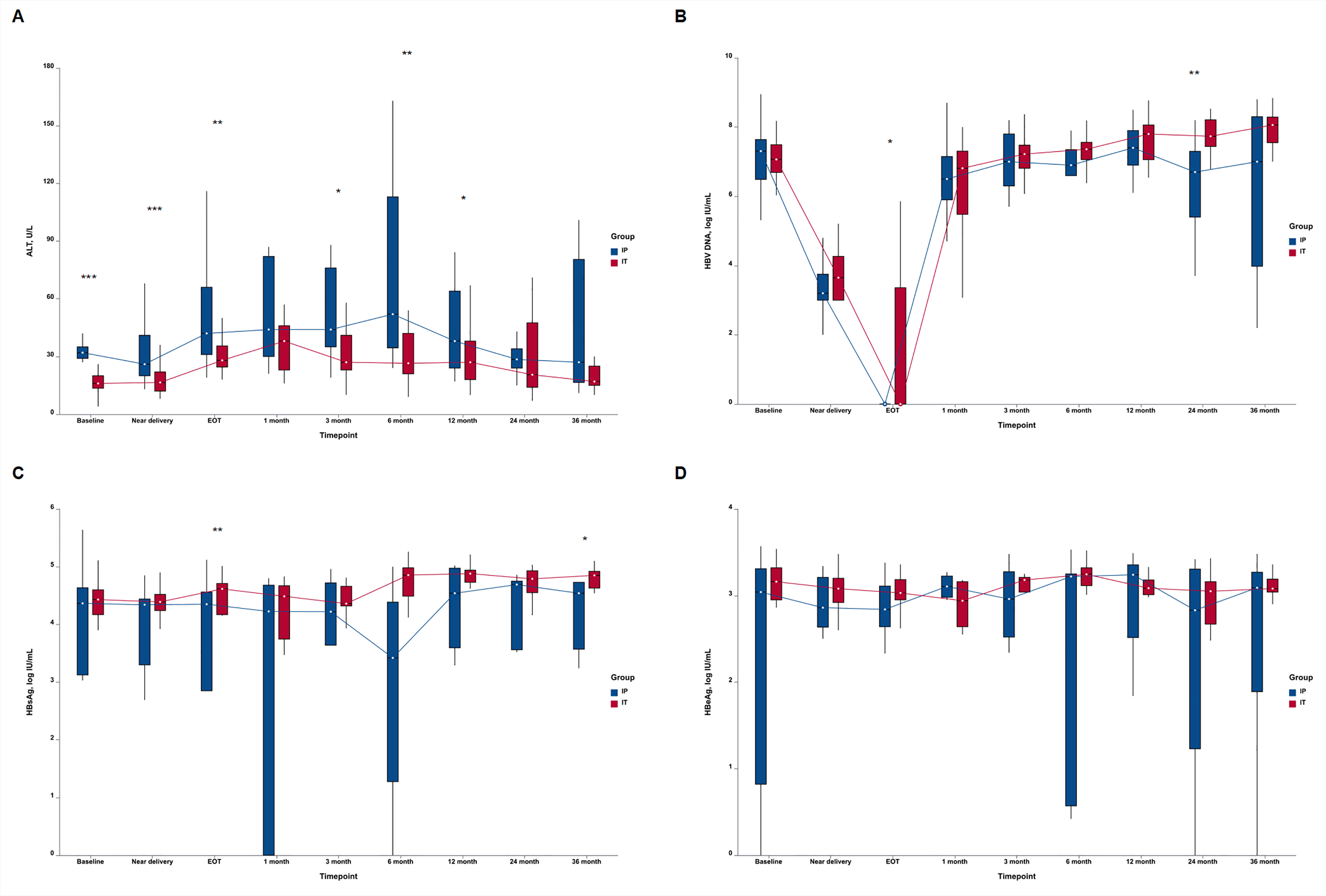


**Fig. S3 The comparison of ALT and virologic markers between participants with immune-tolerance and HBeAg-positive** **indeterminate phase.** A-D described the comparison of ALT, HBV DNA, HBsAg, and HBeAg between participants with immune-tolerance and HBeAg-positive indeterminate phase, respectively. The horizontal axis represents different time points, and the vertical axis represents the levels of variables. The red bars and blue bars represent participants with IT and HBeAg-positive IP, respectively. p-values < 0.05, 0.01, and 0.001 was marked by *, **, and ***, respectively. ALT, alanine aminotransaminase; DNA, deoxyribonucleic acid; EOT, end of treatment; HBeAg, hepatitis B e antigen; HBsAg, hepatitis B surface antigen; HBV, hepatitis B virus; IP, indeterminate phase; IT, immune-tolerance.


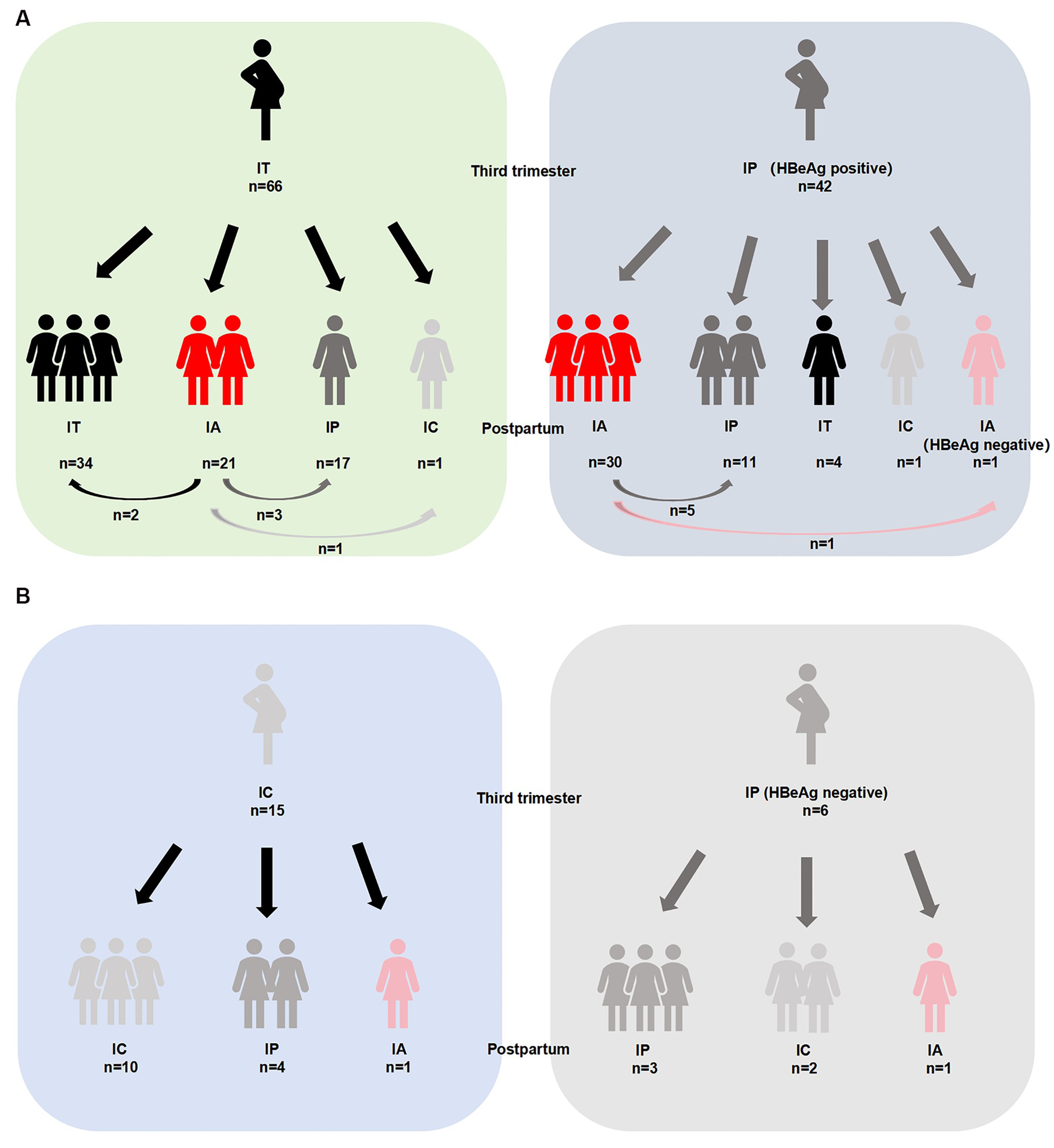


**Fig. S4 The postpartum dynamics of natural history among participants with different phase.** A and B described the dynamics of natural history among HBeAg-positive and HBeAg-positive pregnant women, respectively. Different color indicated different phases. HBeAg, hepatitis B e antigen; IA, immune-active; IC, inactive carrier; IP, indeterminate phase; IT, immune-tolerance.
